# Incidence of microcarcinoma and non‐microcarcinoma in ultrasound‐found thyroid nodules

**DOI:** 10.1186/s12902-021-00700-1

**Published:** 2021-03-04

**Authors:** Zhi Chen, Singla Sethiel Mosha, Tong Zhang, Ming Xu, Yanli Li, Zhuoqing Hu, Weiqiang Liang, Xiaoyi Deng, Tingting Ou, Ling Li, Wangen Li

**Affiliations:** 1 Department of Endocrinology, The Second Affiliated Hospital, Guangzhou Medical University, No.250 Changgang Road East, Haizhu District, 510260 Guangzhou, China; 2 Department of Endocrinology, The Third Affiliated Hospital, Southern Medical University, No.183 Zhongshan Avenue West, Tianhe District, 510630 Guangzhou, China; 3 Department of Medical Ultrasound, The Second Affiliated Hospital, Guangzhou Medical University, No.250 Changgang Road East, Haizhu District, 510260 Guangzhou, China; 4 Department of Endocrinology, Affiliated Nanhai Hospital of Southern Medical University, No.40 Foping Road, Nanhai District, 528200 Foshan, China

**Keywords:** Thyroid nodules, Ultrasound, Carcinomas, Screen

## Abstract

**Backgrounds:**

The incidence of thyroid nodules is increasing year by year around the world. However, ultrasound is not recommended as a screening test for the general population or patients with a normal thyroid on palpation by the American Association of Clinical Endocrinologists (AACE). In practice, some individuals with normal thyroid palpation have nodules that can just be found out by ultrasound. No studies have directly described the risk of nodules found by ultrasound or by palpation up to now. More evidence is needed to carry out for helping us balance the over diagnosis and missed diagnosis of malignant lesions. Therefore, we carried out a retrospective study to investigate the incidence of malignant lesions in ultrasound-found nodules in a large cohort.

**Methods:**

We conducted a retrospective analysis involving 2957 patients who underwent thyroid ultrasound evaluation and fine-needle aspiration (FNA) between Jan 2013 and Dec 2019. The cytologic examinations were analyzed based on the Bethesda system. For nodules suspected to be follicular neoplasm or other malignant tumors by cytological tests, patients were recommended for surgery and histopathology examinations.

**Results:**

Compared with palpation-found nodules, ultrasound-found nodules were presenting less as purely cystic nodules (10.1 % vs. 39.9 %, x^2^ = 355.69, p = 0.000), smaller size (17.5 ± 9.9 mm vs. 28.0 ± 12.5 mm, t = 23.876 p = 0.000), and higher TI-RADS score (5.5 ± 2.9 vs. 3.4 ± 3.3, t = 18.084, p = 0.000), respectively. More ultrasound-found nodules were diagnosed as carcinoma by histology examinations [136 (11.2 %) nodules found by ultrasound vs. 68 (3.9 %) by palpation, x^2^ = 59.737, p = 0.000], and 88 (64.7 %) nodules found by ultrasound were non-microcarcinoma. Among the malignant nodules confirmed by histopathology, a higher proportion of microcarcinoma was detected in ultrasound-found nodules [35.3 % (48/136) vs. 16.2 % (11/68), x^2^ = 8.183, p = 0.004].

**Conclusions:**

In view of the results observed in our research, malignant nodules were more common in nodules screened out by ultrasound, and nearly two thirds of them were non-microcarcinoma. We suggest the recommendation against screening thyroid nodules by ultrasound needs to be re-evaluated.

## Background

The incidence rate of thyroid nodules has an annual increasing trend worldwide. The overall prevalence rate was 49 %-68 % in general population of China, Europe and America by using ultrasound [1–3]. As thyroid nodules are usually the first sign of cancer, the primary goal of treatment is to distinguish between malignant and benign lesions.

In practice, more and more physicians took thyroid ultrasound as the preferred examination because of its noninvasive and inexpensive. However, recent researches reported that screening for thyroid cancer has led to a significant increase in the global diagnosis rate of the disease, but no change in mortality [4, 5]. Therefore, the American Association of Clinical Endocrinologists (AACE) does not recommend ultrasound as a screening test for the general population or patients with a normal thyroid on palpation and a low clinical risk of thyroid disease [6]. Similarly, The United States Preventive Services Task Force recommends against screening for thyroid cancer in asymptomatic adults [7]. Nevertheless, the AACE prescription standard for ultrasound screening is only at a level 4 evidence and GRADE C recommendation, which means it is based on expert experience with no conclusive risks or benefits [6]. We believe that it is necessary to evaluate the nodules that can only be detected by ultrasound. Therefore we can decide whether it is appropriate to carry out ultrasound examination for patients who do not have nodules detected by palpation.

Up to now, no studies have directly compared the risk of ultrasound-found and palpation-found nodules. More researches evidence is needed to carry out for accumulating evidence to help us balance the over diagnosis and missed diagnosis of malignant lesions.

The purpose of this retrospective study was to investigate the incidence of malignant lesions in ultrasound-found nodules.

## Methods

### Patients

We conducted a retrospective analysis involving 2957 patients who underwent thyroid ultrasound evaluation and fine-needle aspiration (FNA) between January 2013 and December 2019. Patients were divided into two groups according to the detection way of the nodules. The ultrasound-found group refers to nodules discovered by ultrasound examination. The palpation-found group means that the nodules were found by patients themselves or by physicians when performing physical examination.

### Ultrasound evaluation

Risk of nodules was reported by ACR thyroid imaging, reporting and data system (TI-RADS) [8]. The characteristics of thyroid nodules were evaluated from five categories: composition, echogenicity, shape, margin, and echogenic foci. Each category has a score, and a total score was obtained by adding the five scores, which is the TI-RADS score. The size of nodules was expressed by the maximum diameter.

### Cytology and histology examinations

FNA was performed by a conventional method, and at least two samples were taken per nodule. All our FNA samples were diagnosed at Guangzhou Kingmed Diagnostics which is the first pathology laboratory certified by the College of American Pathologists in China [9]. Cytology reports were based on the Bethesda system [10]. Six diagnostic categories include: (I) non-diagnostic or unsatisfactory, (II) benign, (III) atypia or follicular lesion of undetermined significance, (IV) follicular neoplasm, (V) suspicious for malignancy and (VI) malignant. We recommended surgery for patients with the last three categories of cytological reports and obtained corresponding histologic reports.

### Statistical analysis

All grouped data in accordance with normal distribution were described by mean ± standard deviation. The unpaired *t* test was used to compare the mean nodule size and TI-RADS score between ultrasound-found and palpation-found groups. The comparison of incidence for categorical data between groups was analyzed by chi-square test. Statistical analyses were performed using SPSS Statistics for Windows ver. 18.0. Statistical significance was defined if p < 0.05.

## Results

### Characteristics of patients

The characteristics of two groups of patients were shown in Table 1. Age, gender, course of disease, and body mass index (BMI) of patients were compared. There were no differences between two groups.

**Table 1:** Characteristics of patients

	Ultrasound-found (*n*=1212)	Palpation-found (*n*=1745)
Male (%)	296 (24.4)	329 (18.9)
Age (years)	48.3±13.1	48.0±14.5
Course of disease (months)^a^	2 (0.1, 240)	3 (0.1, 480)
BMI (kg/m^2^)	23.1±3.7	22.5±2.9

^a^Median (range)

### Characteristics of two types of nodules

In the group of ultrasound-found nodules, less purely cystic nodules were presented (10.1 % vs. 39.9 %). The mean diameter of ultrasound-found nodules was 17.5 ± 9.9 whereas that of palpation-found nodules was 28.0 ± 12.5. A higher TI-RADS score was observed in the group of ultrasound-found nodules. The details were shown in Table 2.

**Table 2:** Characteristics of two types of nodules

	Ultrasound-found (*n*=1212)	Palpation-found (*n*=1745)	X^2^/t value	*P*value
Purely cyst (n/%)	122 (10.1)	697 (39.9)	335.69	0.000
Size (mm)	17.5±9.9	28.0±12.5	23.876	0.000
TI-RADS score	5.5±2.9	3.4±3.3	18.084	0.000

### Incidence of thyroid carcinoma on histology

**Fig. 1:**
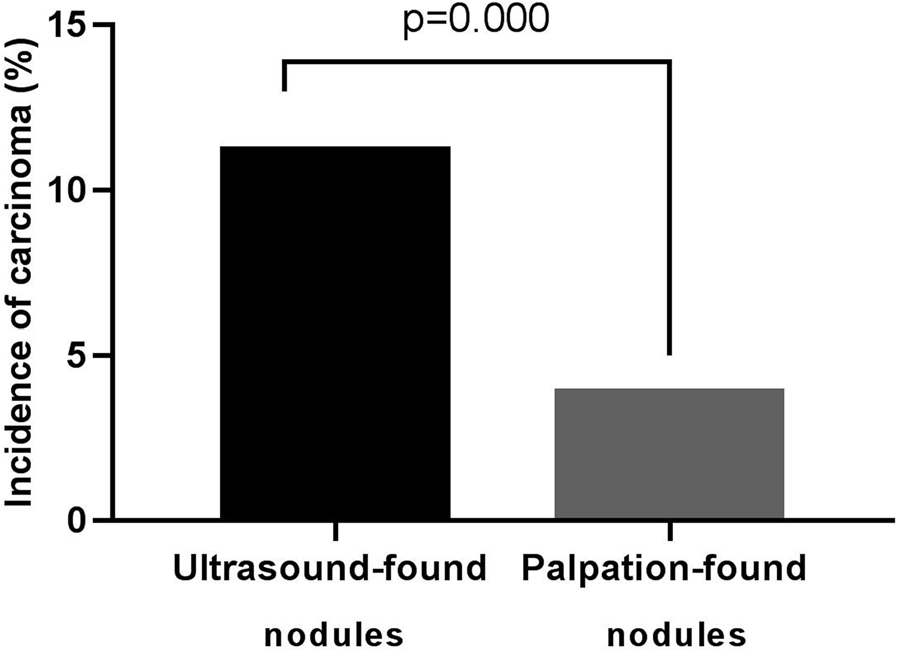
Comparison of the incidence of thyroid carcinoma between ultrasound group and palpation group

A higher proportion of malignant nodules were confirmed by histopathology in ultrasound group. As shown in Fig. 1, a total of 136 (11.2 %) of ultrasound-found nodules and 68 (3.9 %) of palpation-found nodules were diagnosed as carcinoma (x^2^ = 59.737, *p* = 0.000).

### Proportion of microcarcinoma and non‐microcarcinoma

**Fig. 2:**
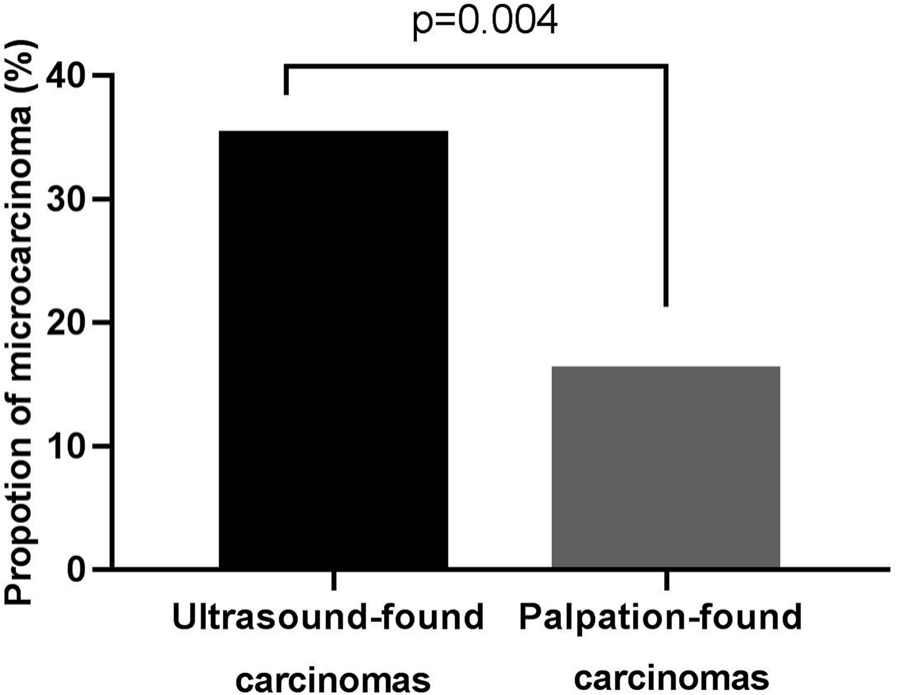
Comparison of the proportion of microcarcinoma between ultrasound group and palpation group

Among carcinomas in ultrasound group, 35.3 % (48/136) nodules were microcarcinomas (with a diameter smaller than 1 cm) and 64.7 % (88/136) were non-microcarcinomas (with a diameter greater than 1cm). More microcarcinomas were detected in malignant nodules found by ultrasound. 35.3 % (48/136) ultrasound-found carcinomas and 16.2 % (11/68) palpation-found ones were micro-carcinomas (x2 = 8.183, *p* = 0.004), respectively (Fig. 2).

## Discussion

Our study indicated that ultrasound-found nodules presented a greater malignancy risk than palpation-found ones. There were several explanations for the results. First, only 10 % of ultrasound-found nodules were purely cystic, which are highly likely to be benign [6, 11]. In contrast, purely cystic nodules were nearly 40 % in palpation-found ones. Second, ultrasound-found nodules were smaller in size and they had higher TI-RADS score than palpation-found ones.

In our study, the evaluation of ultrasound features of nodules was based on TI-RADS published by ACR in 2017 [8]. In ACR TI-RADS, the characteristics of thyroid nodules were evaluated from five features. Each feature has a score, and a total score was obtained by adding the five scores. The total score determined the TI-RADS category of nodules, ranging from 1 to 5. In the research of Kwak et al. [12], the counting method was used rather than weighting method. The counting method is to summarize the number of suspicious ultrasound features, regardless of the weight of malignant possibility of the features. Recently, Zhou et al. [13] published a multicenter study on Chinese population, which verified the diagnostic efficacy of the newly established Chinese TI-RADS (C-TIRADS). C-TIRADS also adopts counting method, which has good sensitivity and specificity. In our study, taking into account the familiarity with the criteria of medical and ultrasound staff, we used ACR TI-RADS to evaluate the nodules. Statistical analysis shows that there is no difference in diagnostic performance between weighted method and counting method [13, 14]. If we take other TI-RADS for statistical analysis, the results may not be different from the current ones.

Unlike palpation-found nodules, ultrasound-found nodules were smaller and often located deep in the thyroid tissue. Hence the relation of the nodule size between malignancy risk and prognosis as recommended by AACE became controversial [2].

Though some studies found that malignant risk is associated with nodule size, for example, a series of observational studies have found that thyroid cancers over 4 cm were associated with more aggressive behavior whereas tumors smaller than 1.5 cm had a good overall prognosis [15–17]. However, some other researches indicated no correlation between size and risk [18, 19]. A recent study found that the impact of nodule size on the malignancy risk differed according to the ultrasound pattern. A large nodule size (≥ 3 cm) showed a higher malignancy risk than smaller nodules in intermediate- and low-suspicion nodules [20].

We are convinced that our study findings as demonstrated above, highlight a conflict between AACE recommendations and clinical practice in the following aspects: AACE’s recommends not to conduct ultrasound screening for thyroid nodules, while the recommendation of FNA was based on nodule size [6]. American Thyroid Association (ATA) also recommends FNA based on nodule size [11]. In our study, the average diameter of nodules detected by ultrasound was 1.75 cm, which was within both of the recommended range of FNA.

Another conflict between AACE recommendations and recent reality highlighted in this study is that AACE recommends against ultrasound screening based on a significant increase in global thyroid cancer prevalence but a constant mortality rate [5, 6]. However, latest published study found that in America, incidence-based thyroid cancer mortality rose from 0.40 to 100,000 person-years in 1994–1997 to 0.46 per 100,000 person-years in 2010–2013 [21]. In China, the mortality increased from 0.30 to 100,000 in 2005 to 0.35 per 100,000 in 2015 [22].

Under current guidelines, only a small portion of patients could be under active surveillance for microcarcinoma [23]. Furthermore, in our study of ultrasound-found nodules, there were as high as two thirds that were non-microcarcinoma. For these nodules larger than 1 cm, it is generally believed that the benefits of surgery outweigh the risks [6].

The limitation of this study is that ultrasound evaluations were performed by different operators and machines, so bias may exist.

## Conclusions

In summary, since malignant nodules were more common in the ultrasound-found nodules, and nearly two thirds of which were non-microcarcinoma, we suggest the recommendation against screening thyroid nodules by ultrasound needs to be re-evaluated.

## Data Availability

The datasets used and/or analysed during the current study are available from the corresponding author on reasonable request.

## Abbreviations

AACE: American Association of Clinical Endocrinologists
ACR: American College of Radiology
ATA: American Thyroid Association
FNA: fine-needle aspiration
TI-RADS: Thyroid imaging reporting and data system
